# Prediction of liver toxicity and mode of action using metabolomics in vitro in HepG2 cells

**DOI:** 10.1007/s00204-017-2079-6

**Published:** 2017-09-30

**Authors:** Tzutzuy Ramirez, Alexander Strigun, Andreas Verlohner, Hans-Albrecht Huener, Erik Peter, Michael Herold, Natalie Bordag, Werner Mellert, Tilmann Walk, Michael Spitzer, Xiaoqi Jiang, Saskia Sperber, Thomas Hofmann, Thomas Hartung, Hennicke Kamp, Ben van Ravenzwaay

**Affiliations:** 1 BASF SE Experimental Toxicology and Ecology, Carl-Bosch Str.38, 67056 Ludwigshafen Am Rhein, Germany; 2 Metanomics GmbH, Tegeler Weg 33, 10589 Berlin, Germany; 3 Johns Hopkins Bloomberg School of Public Health, Center for Alternatives to Animal Testing (CAAT), Baltimore, MD USA; 4 CAAT-Europe, University of Konstanz, Konstanz, Germany

**Keywords:** Metabolomics, Liver toxicity, In vitro, HepG2 cells

## Abstract

Liver toxicity is a leading systemic toxicity of drugs and chemicals demanding more human-relevant, high throughput, cost effective in vitro solutions. In addition to contributing to animal welfare, in vitro techniques facilitate exploring and understanding the molecular mechanisms underlying toxicity. New ‘omics technologies can provide comprehensive information on the toxicological mode of action of compounds, as well as quantitative information about the multi-parametric metabolic response of cellular systems in normal and patho-physiological conditions. Here, we combined mass-spectroscopy metabolomics with an in vitro liver toxicity model. Metabolite profiles of HepG2 cells treated with 35 test substances resulted in 1114 cell supernatants and 3556 intracellular samples analyzed by metabolomics. Control samples showed relative standard deviations of about 10–15%, while the technical replicates were at 5–10%. Importantly, this procedure revealed concentration–response effects and patterns of metabolome changes that are consistent for different liver toxicity mechanisms (liver enzyme induction/inhibition, liver toxicity and peroxisome proliferation). Our findings provide evidence that identifying organ toxicity can be achieved in a robust, reliable, human-relevant system, representing a non-animal alternative for systemic toxicology.

**Electronic supplementary material:**

The online version of this article (doi:10.1007/s00204-017-2079-6) contains supplementary material, which is available to authorized users.

## Introduction

Toxicology is undergoing a paradigm shift, from predominantly observational science (based on animal testing), to predominantly predictive science focusing on target-specific, mechanism-based, biological observations, contingent upon in vitro data and in silico predictions, often referred to as toxicology for the twenty-first century (Hartung 2009). The development and application of modern tools can provide deeper insights into the molecular mechanisms underlying toxicity in a high throughput manner (Attene-Ramos et al. 2015; Liu et al. 2015). Such developments are being driven by the need to improve the safety evaluation of chemicals in a more efficient, human-relevant context (Judson et al. 2014) to meet changing regulations and promote the use of non-animal models to predict toxicity (Ramirez et al. 2013b).

Generally, toxicity studies require large numbers of animals, take several months to years to complete, are usually very costly, and can only test low numbers of compounds in a given time period. Current animal testing is primarily performed in rats and mice, and although these rodents exhibit many of the same responses to chemicals as humans, there are qualitative and particularly quantitative differences. Most toxicology studies, particularly those used to fulfil regulatory requirements rely on apical endpoints, such as signs of clinical toxicity, hematology, urinalysis as well as clinical and histopathological evaluations. Despite these numerous evaluations, their main target is to determine a dose with no effect (no observed effect level: NOEL), rather than to understand the mechanisms responsible for inducing toxicity. The latter, however, is an essential component to address questions about the human relevance of these animal tests. The answer to such questions is conventionally circumvented by introducing safety factors (usually ranging between 100 and 1000-fold below the observed effect level). The appropriateness of these safety factors is hardly ever addressed. New approaches to toxicity testing offer the chance to open this “black box” of unknown liabilities, and provide a valuable foundation for more targeted risk assessment. Experience from clinical trials suggests that 20–40% of drugs fail because of toxic side-effects not predicted (Arrowsmith 2012), about half of this being liver toxicities, and only about 43% of these predictable in retrospect from the rodent studies (Olson et al. 2000), which are the only information typically generated for industrial chemicals.

Metabolomics can provide a readout of a biological system’s biochemical and physiological status (Choucha Snouber et al. 2013; Van den Hof et al. 2014; Vermeersch et al. 2015). In comparison to other omics technologies, it is thought to best represent a phenotype and hence “classical toxicology” (Bouhifd et al. 2013). Metabolome analyses of body fluids such as urine or blood plasma have been shown to provide new insights into toxicity (Kamp et al. 2012a; Mattes et al. 2014; Reily and Tymiak 2015) as well as predicting the toxicity of compounds at an early stage of development (van Ravenzwaay et al. 2012a). Such technologies are not only highly useful to gain more information from animal studies but also help to reduce animal testing by refining the methods. However, the above-mentioned investigations necessarily still rely on animal studies and have a limited potential to investigate the cellular, mechanistic origin of toxicity in humans.

Therefore, we decided to apply metabolomics in an in vitro human cell system, to address whether organ toxicity could be identified in a robust and reproducible way. Here, we report on a concept we have developed using a highly reproducible HepG2 liver cell-based system validated with 35 test substances (Table 1, Supplementary Table 1) over a period of more than 3 years using both supernatant and intracellular metabolome analysis of natural low-molecular-weight endogenous constituents of cells (Ramirez et al. 2012, 2013a).

**Table 1:** Overview of the test substances used for treatment of HepG2 cells for 48 h

Substance	CAS-Nr.	Chemical class	Category	MoA (target in)
4-Chloroaniline	106-47-8	Amine	Industrial chemical	Methemoglobin formation
**β-Naphthoflavone**	**6051-87-2**	**Benzoflavone**	**Industrial chemical**	**Liver enzyme inducer**
Acetaminophen	103-90-2	Drug	Pharma	Cyclooxygenase inhibitor
**Acifluorfen**	**50594-66-6**	**Diphenylether**	**Herbicide**	**Inhibition of protoporphyrinogen oxidase (PPO)**
**Aroclor 1254**	**11097-69-1**	**Polychlorinated biphenyl**	**Industrial chemical**	**Liver enzyme inducer**
Benzylbutyl phthalate	85-68-7	Phthalic acids	Industrial chemical	Peroxisome proliferation
**Bezafibrate**	**41859-67-0**	**Fibric acids**	**Hypolipidemic agents**	**Peroxisome proliferation**
Carbaryl	63-25-2	Carbamate	Insecticide	Acetylcholinesterase (AChE) inhibitors
Cyclosporin A	59865-13-3	Peptides, cyclic	Immunosuppresive agents	Block the transcription of cytokine genes in activated T cells
Cycloxidim	101205-02-1	Pyrans	Herbicide	Fatty acid biosynthesis in grass
**Dichlorprop**	**120-36-5**	**Phenoxyacetate**	**Herbicide**	**Action like indole acetic acid (synthetic auxins)**
**Dichlorprop-p**	**15165-67-0**	**Phenoxyacetate**	**Herbicide**	**Action like indole acetic acid (synthetic auxins)**
Digitoxin	71-63-6	Digitalis glycosides	Anti-arrhythmia agent	Heart/Na–K ATPase inhibitor
**Dimethenamide**	**87674-68-8**	**Chloroacetamide**	**Herbicide**	**Long chain fatty acid inhibitor**
**Dimethenamide-p**	**163515-14-8**	**Chloroacetamide**	**Herbicide**	**Long chain fatty acid inhibitor**
Dimethoate	60-51-5	Organophosphate	Insecticide	Acetylcholinesterase (AChE) inhibitors
Dimethylformamide	68-12-2	Formamide	Industrial chemical	Not applicable (liver toxicant)
**Fipronil**	**120068-37-3**	**Phenylpyrazole**	**Insecticide**	**GABA -gated chloride channel blockers**
**Fluoroglycofen-ethyl**	**77501-90-7**	**Diphenylether**	**Herbicide**	**Inhibition of protoporphyrinogen oxidase (PPO)**
Fluoxetine hydrochloride	56296-78-7	Propyolamine	Antidepressant	Selective serotonin reuptake inhibitor
Imazamox	114311-32-9	Imidazole	Herbicide	Inhibition of acetolactate synthase ALS (acetohydroxyacid synthase AHAS)
MCPA	94-74-6	Phenoxyacetate	Herbicide	Action like indole acetic acid (synthetic auxins)
**Mecoprop**	**93-65-2**	**Phenoxyacetate**	**Herbicide**	**Action like indole acetic acid (synthetic auxins)**
**Mecoprop-p**	**16484-77-8**	**Phenoxyacetate**	**Herbicide**	**Action like indole acetic acid (synthetic auxins)**
**Metconazole/cis**	**115850-27-6**	**Triazole derivative**	**Fungicide**	**Enzyme inhibitor**
**Metconazole/cis–trans**	**125116-23-6**	**Triazole derivative**	**Fungicide**	**Enzyme inhibitor**
Nicosulfuron	111991-09-4	Sulfonylurea	Herbicide	Inhibition of acetolactate synthase ALS (acetohydroxyacid synthase AHAS)
**Pendimethalin**	**40487-42-1**	**Dinitroaniline**	**Herbicide**	**Microtubule assembly inhibition**
Phenobarbital sodium salt	57-30-7	Barbiturate	Sedative	Brain/GABA modulator
Pentobarbital sodium salt	57-33-0	Barbiturate	Sedative	Brain/GABA modulator
Pyridaben	96489-71-3	Pyridazine	Pesticide	Mitochondrial complex I electron transport inhibitors
Tamoxifen	10540-29-1	Stilbenes	Antineoplastic	Estrogen receptor modulator
Tetracycline	60-54-8	Tetracyclines	Pharma	Protein synthesis inhibitor
Verapamil hydrochloride	152-11-4	Phenethylamine	Anti-arrhythmia agent	Heart/Ca2 + channel blocker, CYP3A4 inhibitor
**Vinclozolin**	**50471-44-8**	**Oxazoles**	**Fungicide**	**NADH cytochrome c reductase in lipid peroxidation**
**Wy-14643**	**50892-23-4**	**Pyrimidines**	**Hypolipidemic agents**	**Peroxisome proliferation**

Those highlighted in bold represent compounds discussed in the text

## Materials and methods

### Cell culture

HepG2 (human hepatocyte carcinoma, acquired from ATCC, clone HB8065, maximum passage number 20) cells were maintained and grown on Dulbecco’s MEM media supplemented with 1 v/v% of penicillin/streptomycin, l-glutamine (200 mM, 1 v/v%), non-essential amino acids (100x, 1 v/v%) and 10% FBS (Biochrom, Germany). For experiments, 0.45 × 10^6^ cells were grown on multi-well plates or lumox^®^ dishes 35 (35 mm, Sarstedt, Germany) and incubated under 5% CO_2_ at 37 °C for 24 h (Bordag et al. 2016b). After incubation, culture media were exchanged and chemical treatment was applied for 48 h. Cells and their supernatants were then harvested, frozen and stored at − 80 °C under inert gas atmosphere until analysis. Cell viability was measured by WST-1, cells were seeded in dishes and treated as well as the cells used for metabolome analysis. After exposure time, cell culture media was removed and 500 µL of the WST-1 working reagent per dish were added and dishes were incubated at 37 °C. After 1 h, 100 µL of supernatant were transferred to a 96-well plate in duplicates. Absorbance was measured at 450 nm with a reference wavelength of 600–700 nm.

### Treatment substances

The substances used for the experiments reported here have been selected because of their known in vivo effects. In particular, they have been chosen to proof whether HepG2-based in vitro metabolomics can serve as a tool for the detection of different liver toxicities. Therefore, these compounds were selected based on the knowledge about their liver effects including the underlying modes of action. The following test substances were selected for treatment of HepG2 cells in different experiments (Table 1).

### Range finder experiments

Prior to metabolome experiments, range finder experiments for all tested substances were performed to select a concentration range at which the protein concentration was not reduced below 80% compared to controls, and preferably in the range of 90% at the highest concentration. For treatment with each test substance, increasing concentrations were set up (in triplicates). After 48 h, protein content was measured using bicinchoninic acid (BCA; see below). The concentration that reduced the total protein content by a maximum of 20% was designated as the high dose (HD) for the main experiment. In general, one-third of the HD concentration was selected as the low dose (LD).

### Treatment tests

For treatment, dishes were treated with vehicle control only (VC, final concentration of DMSO was 0.5%, 16 replicates each time), or with HD test substance (final concentration of DMSO was 0.5%, 8 replicates each time) and LD test substance (final concentration of DMSO was 0.5%, at least 8 replicates each time). In addition, blank controls were set up as dishes without cells but containing media (16 replicates) and technical replicates (pools) were prepared as samples containing only cells with VC (0.5% DMSO, 16–20 replicates per testing of 2 test substances). After treatment, supernatant and cells were harvested, strictly ensuring that the time for harvesting every sample did not exceed 30 s.

For exosome analysis, cell supernatants (1 mL per sample only) were transferred to Eppendorf tubes, quickly centrifuged to eliminate potential cell debris, re-transferred to fresh Eppendorf tubes, gassed with argon to avoid sample oxidation and stored at − 80 °C until measurement. For the analysis of intracellular metabolomics, the bottom of the dishes was removed with a scalpel and rinsed three times in 0.9% NaCl solution (pre-warmed to 37 °C). After rinsing, membranes were transferred to pre-cooled 2 mL Eppendorf™ tubes (placed in liquid nitrogen). The Eppendorf tubes were then placed in dry ice and quenched with 600 µL of dichloromethane-ethanol (DCM/EtOH, 9:11, v/v at − 80 °C). Every sample was gassed with argon as with the supernatants. Samples were stored at − 80 °C until further processing. Further details on the preparation of the metabolome samples can be found in Bordag et al. (2016a).

### Determining protein content

Protein content was determined in a sister culture handled and treated exactly as the cultures used for metabolome analysis. Three replicates per test substance concentration or control were prepared and grown in dishes. After 24 h of seeding, cells were treated for 48 h. After treatment, cells were lysed with 0.1% triton x-100 (Sigma-Aldrich, Germany). Lysates were pipetted (25 µL) into 96-well plates and incubated with 200 µL of BCA solution for 30 min at 37 °C. After incubation, plates were measured with a photometer and protein content was calculated by normalization with a standard curve (Pierce, Thermo Fisher, Germany).

### Analytics: MxP^®^ broad profiling

An extraction method for polar metabolites from cells grown on dishes (Balcke et al. 2011) was modified to comprehensively extract lipid and polar metabolites. For this new extraction protocol 4 mg of ammonium acetate dissolved in 10 µL water, 400 µL water, 50 µL toluene and 45 µL methyl tert-butyl ether were added, containing internal standards for MxP^®^ Broad Profiling as described previously (van Ravenzwaay et al. 2007). To each sample, 3 mm stainless steel beads were added and the samples homogenized with an Omni Bead Ruptor 24 3 times for 30 s each (10 s pause in between) at 3.5 m/s. The extracts were transferred to Ultrafree ^®^—MC Durapore PVDF 5 µm filter units (Millipore UFC30SV00) and spun down for 5 min at 12,000 rpm, 12 °C in an Eppendorf™ 5417R microcentrifuge. Filter units were discarded, 200 µL DCM were added to the filtrates, agitated for 5 min at 1400 rpm, 12 °C in an Eppendorf™ Thermomixer Comfort and phase separation was achieved by centrifugation for 5 min at 12,000 rpm, 12 °C. Subsequently, aliquots of the polar and non-polar fractions were further treated and analyzed as described for MxP^®^ Broad Profiling (Jung et al. 2013) with GC–MS (6890 GC (Agilent) coupled to a 5973 MS-System (Agilent) and LC–MS/MS (1100 HPLC (Agilent) coupled to an API4000 MS/MS-System (Applied Biosystems), using for LC–MS/MS a technology, which allows MRM in parallel to a full scan analysis (Walk and Dostler 2003).

Pooled reference samples derived from aliquots of all control samples (per matrix) were measured in parallel throughout the entire analytical process. Spent medium and intracellular data were normalized against the median in the pool reference samples to give pool-normalized ratios (performed for each sample per metabolite). This compensated for inter- and intra-instrumental variation.

To correct for differences in cell numbers within and between different treatment groups, the data for both spent medium and intracellular metabolite levels were also normalized to the within sample median. The median normalization produced a new set of values $X_{ij}^{\text{med}}$ according to the following formula:

$$X_{ij}^{\text{med}} = \frac{{X_{ij} }}{{{\text{median}}(X_{i} .)}},$$

with $X_{i} . = (X_{i1} ,X_{i2} , \ldots ,X_{im} )$, representing the values from the $i{\text{th}}$ sample.

Here, the index *i* = 1, 2,…, *n* denotes the samples and *j* = 1, 2,…, *m* denotes the metabolites, so that $X_{ij}$ represents the pool normalized ratio of metabolite *j* from the sample *i*.

For intracellular metabolomics analysis, the median of each sample was calculated from 117 known and 77 unknown metabolites. In the case of supernatant medium data the sample median was calculated from 70 known and 19 unknown metabolites. A metabolite is regarded as known if the chemical identity of the metabolite has been determined.

To investigate whether the experimental variability remained stable over time, we calculated the variance of every log-transformed metabolite for both pooled samples (technical replicates) and control samples in each work package. These variances were back-transformed to the linear scale, yielding a relative standard deviation (RSD) using the following formula:

$${\text{RSD}} = 1 - 10^{{ - {\text{sd}}_{ \log } }} .$$

### Metabolite profiling and pair-wise comparison

To generate metabolic profiles for the different treatments, the heteroscedastic *t* test (Welch test) was applied to the log-transformed metabolite data to compare treated groups with their respective controls. The *p* values, *t* values and ratios of corresponding group medians were collected as metabolic profiles and stored in the database MetaMap^®^Tox (van Ravenzwaay et al. 2012b). The metabolite patterns were established applying a 5% significance level. To be able to compare the metabolite profiles in HepG2 cells induced by the different treatments, the similarity between two treatments was determined by the Pearson correlation between their respective *t* value profiles. All pair-wise correlations were calculated.

### Statistical analysis

Metabolite values were log10-transformed for the entire statistical analysis to better approximate a normal distribution. For univariate analysis, linear models (statistical software R (R Development Core Team 2014)) were set up with the factors: substance, dose and work package as well as all interactions. All factors were treated as categorical. For principal component analysis, the log-transformed metabolite data was centered and scaled to unit variance. Scaling to unit variance introduced a common scale for all metabolites independent of their absolute variance. Thereby, the resulting models obtained robustness, i.e., a single or few high-variance metabolites could not dominate them.

## Results

### Metabolite identification and general cytotoxicity effects of test substances

**Fig. 1:**
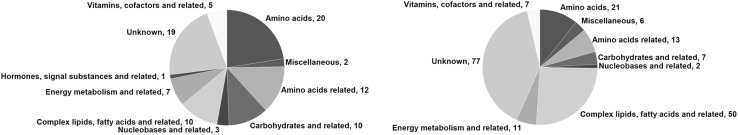
Metabolites distributed according to their metabolite class. Left: 89 metabolites found in the supernatant of HepG2 cells. Right: 194 metabolites found intracellular in HepG2 cells. The distribution and actual numbers of the different identified metabolite classes are depicted, where unknown represents metabolites undergoing chemical class identification

We investigated the analytical capacity of the metabolome platform using state-of-the-art LC–MS/MS and GC–MS, which allowed us to consistently detect, quantify and identify 89 supernatant and 194 intracellular HepG2 cell metabolites (Fig. 1). Although these cells have limitations (i.e., incomplete metabolic competence relative to primary hepatocytes), HepG2 cells represent a well-accepted model of human liver cells simple enough and controllable under in vitro conditions to provide robust data over time.

Prior to selecting HepG2 cells, we investigated several other cellular systems, e.g., precision cut liver slices, HepaRG cells and primary liver microtissues. However, in our experimental set up, which was the same as described here, none of these alternative cellular systems provided reproducible data. The precision cut liver slices had a rather low viability, and a profound difference between each slice, making reliable and consistent metabolomic analyses impossible. The HepaRG cells were obtained in an undifferentiated state, and even after differentiation in vitro using 1% DMSO, analysis of several differentiation markers indicated that the process was not fully reproducible, making the system labile and unsuitable for metabolomics. When applying metabolomics to fresh liver microtissues, the amount of biological material obtained in each microtissue was not sufficient to reliably measure metabolites, i.e., many of the metabolites were below the limit of detection/quantitation. Pooling of microtissues only partly solved this problem and still led to metabolite amounts that were not always sufficiently high for a reliable quantification. A further increase of microtissues to be combined was not considered because the resources needed would render this experimental set-up as unpractical. To our experience, a minimum number of 2 million cells should be used for intracellular metabolome measurements to achieve good quantitation and reproducibility of a high number of targeted metabolites.

To ensure that observed metabolome changes were not related to cell death or general cytotoxicity, we evaluated cell viability (water soluble tetrazolium, WST-1), and protein content (BCA). Cell viability was equal to, or above 90%. Despite careful selection of the high concentration, based on the range-finding experiments, at the high concentration (HC) of 8 out of 33 test substances reduced protein content slightly below our threshold of ≥ 80% (Supplementary Table 2). Test substances reducing protein content, considered a sign of cytotoxicity, induced consistent changes in certain metabolites, which might serve as internal cellular status markers for cell cytotoxicity in the future. However, the altered “cytotoxicity” metabolites did not hinder identification of metabolite changes related to specific toxicity mechanisms (see below).

### Standardization and reproducibility

To ensure the use of novel technologies in an industrial context and their acceptance by regulatory bodies, it is essential to assess quality parameters such as reproducibility and reliability. During the project, we generated huge numbers of samples and realized early on that every step needed to be highly standardized and technically accurate. The major breakthrough was the sample harvesting; every sample must be quenched and shock frozen within a maximum of 30 s. Moreover, avoiding the need for elaborate normalization processes to account for differences in cell numbers obtained during harvesting, required a new process. Therefore, we used Lumox™ dishes with removable breathable membranes supporting the growth of cells and rapid preparation and extraction of intracellular metabolites, which proved to be one essential element for the success of the study.

Lumox™ dishes have several advantages over cell trypsinization and scratching, which not only generate stress for the cells, but also take longer than 1 min to perform, raising the risk of inducing changes in metabolites related to cell processing rather than treatment. Another important feature of our technology was the normalization strategy. This can be achieved by comparing the protein content of sister cultures, a rather inaccurate approach, or by using statistical normalization to the median over all metabolites for the sample, a strategy with the advantage that the median can be determined in the measured sample rather than a sister culture. While small differences occurred in the control baseline levels, the relative change from control to treated was remarkably stable, as identified in the pair-wise comparison of the respective *t* value profiles (data not shown).

**Fig. 2:**
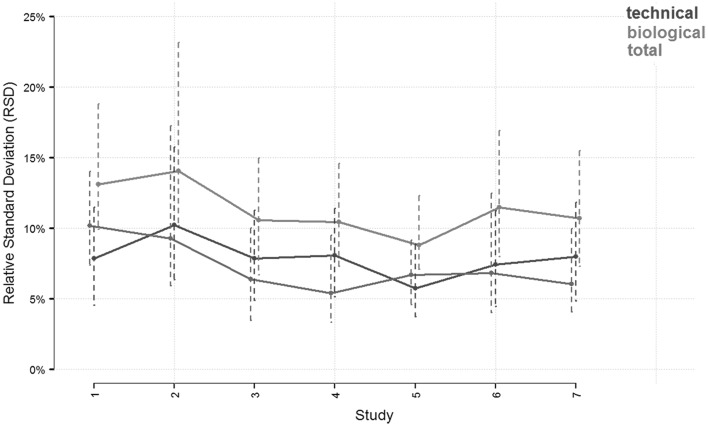
Variability of controls generated over 3 years. Displayed are the total relative standard deviations (RSD) of all control samples grouped per experiment (study) and corrected from the weak variability (depicted in red) and technical (depicted in blue) samples and grouped chronologically according to the time when the experiments were performed. The biological variability (purple) was estimating by subtracting the technical from the total variance on the log-scale and then transforming to RSD as described in the text

The analysis of experimental variability over time demonstrated the robustness and reproducibility of the metabolomics in vitro method, as evaluated by statistical analysis of the metabolite profiles in 1114 cell supernatants and 3556 intracellular samples from 7 experiments performed within 3 years (Fig. 2). Comparing the control samples generated in different work packages revealed a constant behavior of the RSD with small variabilities of about 10–15%, while the technical replicates had an RSD of about 5–10%. Reproducibility was also evaluated under treatment conditions using a reference liver toxic substance, bezafibrate, as a positive control in all experiments. The metabolome profile of different bezafibrate experiments clustered together in all analyses (supernatant/intracellular, low/high dose), indicating the high quality and homogeneity of the samples and experiments.

**Fig. 3:**
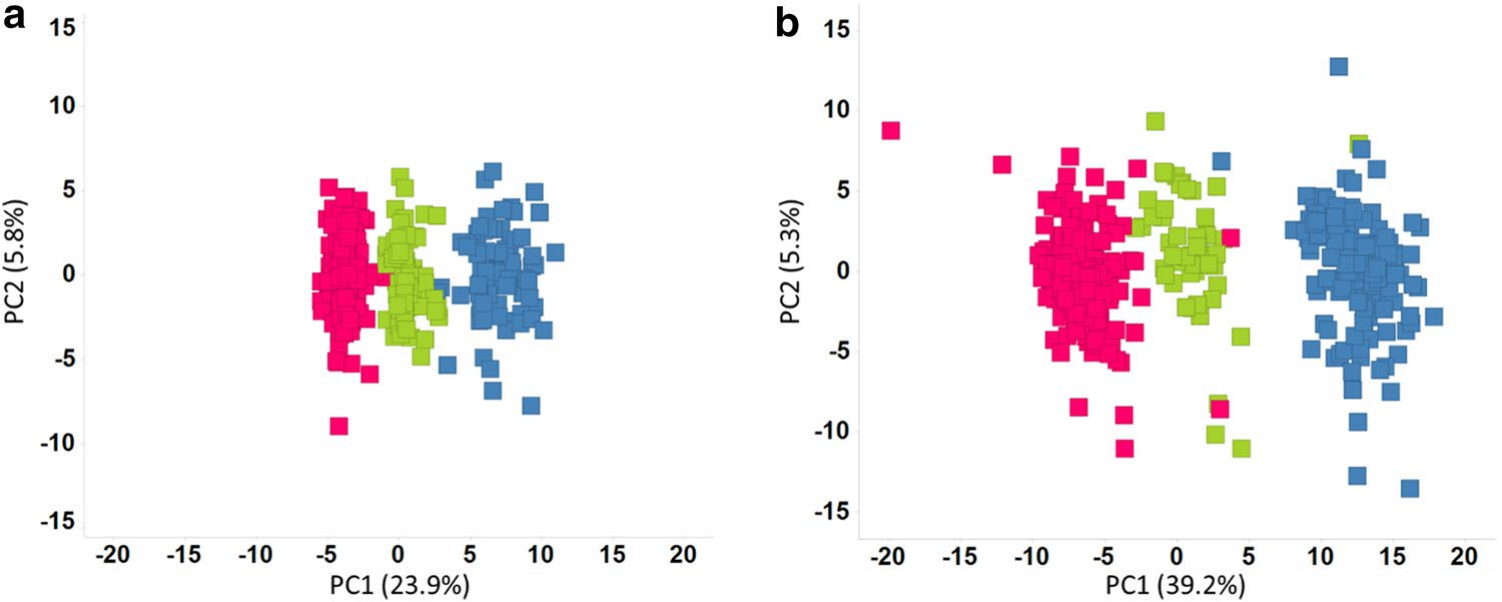
Dose response effects. Principal component analysis (PCA) of all control samples (red) and all bezafibrate treated samples in low dose (LD, green) and high dose (HD, blue) treatments. **a** Supernatant and **b** intracellular metabolomics data (color figure online)

Another important aspect for toxicology is identifying concentration response effects following chemical treatment. Concentration-dependent responses were analyzed in samples treated with bezafibrate by principle component analysis (PCA). The metabolome profile of control, or low and high dose bezafibrate samples clustered together in both supernatant and intracellular samples; however, the samples were all well separated from one another, suggesting observable concentration-dependent effects (Fig. 3).

**Fig. 4:**
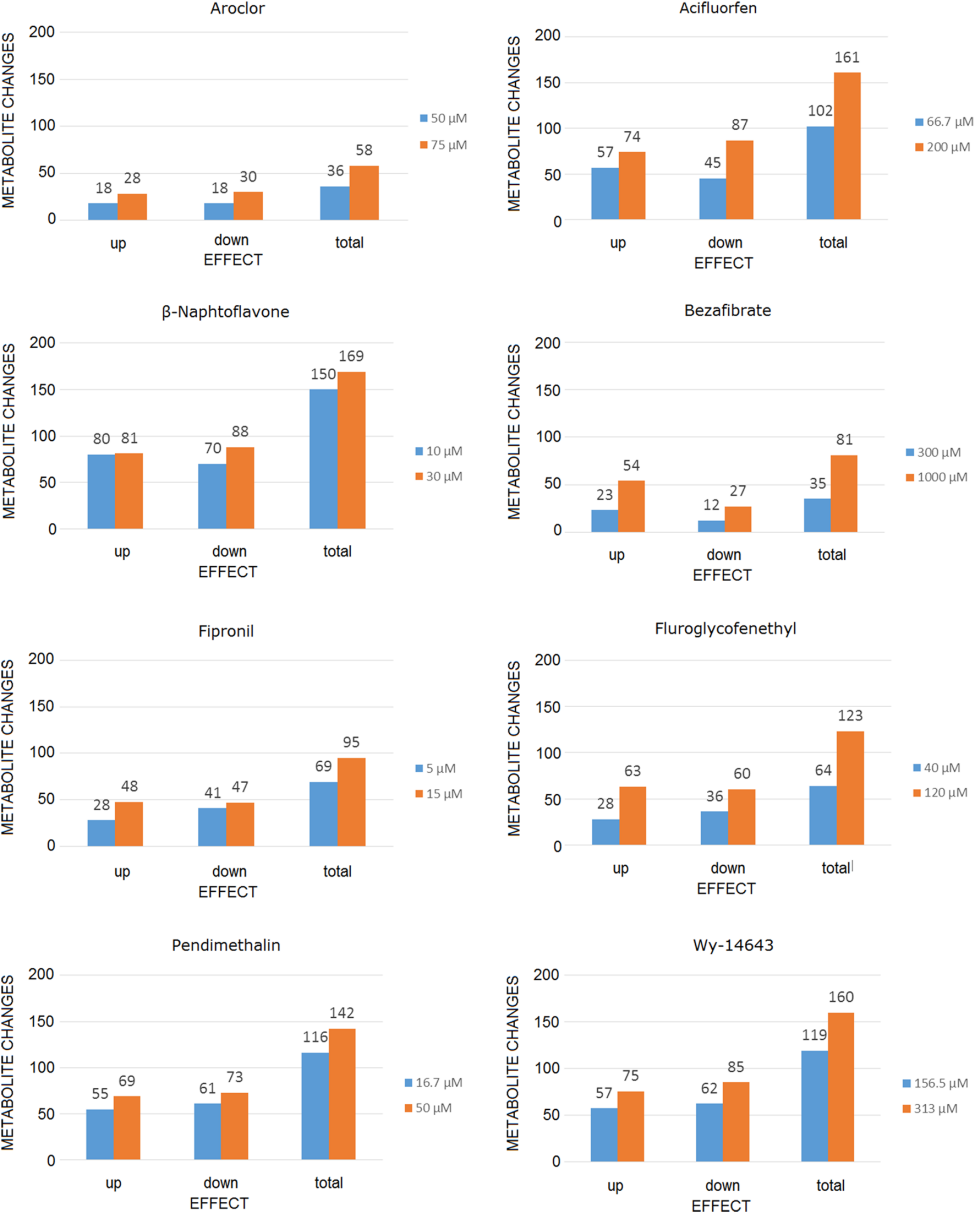
Dose response effects on metabolite changes. The number of changed metabolites induced by liver enzyme inducers and peroxisome proliferators at different doses (low dose in blue and high dose in red, specifications on the dose are depicted per test substance) (color figure online)

In addition to PCA, we also evaluated the number of changed intracellular metabolites after treatment by a small set of the analyzed compounds (Fig. 4). The data revealed dose-dependency in all cases, with higher numbers of changed metabolites recorded after high dose treatments. However, the strength of the dose-dependent effect varied from treatment to treatment. For example, fluoroglycofenethyl exhibited stronger dose-dependency than β-naphthoflavone: the former induced 64 metabolite changes at the low dose and 123 at the high dose level, while the latter already induced 150 metabolite changes at the low dose, which only increased slightly to 169 at the high dose. It is important to note that β-naphthoflavone had much stronger effects at considerably lower concentrations (10 and 30 µM) than fluroglycofenethyl (40 and 120 µM).

Reproducibility was also evaluated for bezafibrate treatments at high dose by means of pair-wise comparison (PWC) using the Pearson correlation ranking. The analysis included a maximum of 14 independent experiments where bezafibrate served as a positive control of liver effects. Previous studies from metabolomics in vivo have indicated that replicates are considered perfectly reproducible when they are top ranking in the PWC over samples or replicates from other conditions (Kamp et al. 2012a). For the PWC analysis, seven independent experiments were available for supernatant, and 14 for intracellular data (both high dose). When analyzing the supernatant, bezafibrate-induced profiles exhibited a high reproducibility, 6/7 experiments occupying the first 6 positions of the Pearson rank, exhibiting a Pearson correlation coefficient (*r*) ranging from 0.94 to 0.85, followed by a cluster of 4 low dose bezafibrate experiments and the 7th high dose experiment (*r* = 0.845, Pearson rank 10). For the PWC of bezafibrate high dose data from the intracellular samples, all ranked in the top 14 positions (*r* = 0.956, Pearson rank 1 to 0.805, Pearson rank 13), followed by bezafibrate low dose and other compounds showing similar toxicity modes of action (data not shown). Since all evaluated experiments covered a period of 3 years, this indicates excellent reproducibility over time.

### Supernatant and intracellular metabolomics of specific liver MoAs

Another major breakthrough was that we were able to measure the metabolites inside the cells. Many metabolomics in vitro studies in mammalian systems only measure metabolites in the cell supernatant. Initially, we also did this before optimizing our standardized process to be rapid enough to reduce intracellular sample variability during harvesting (Bordag et al. 2016a). A further essential aspect of our study was selecting specific reference test substances to monitor specific liver modes of actions (MoAs): one set induced peroxisome proliferation and a second induced the expression of xenobiotic metabolizing liver enzymes, both important modes of action in liver toxicity from in vivo studies. A third set of substances that neither induce liver enzymes nor peroxisome proliferation served as important controls to test the specificity of the system.

**Fig. 5:**
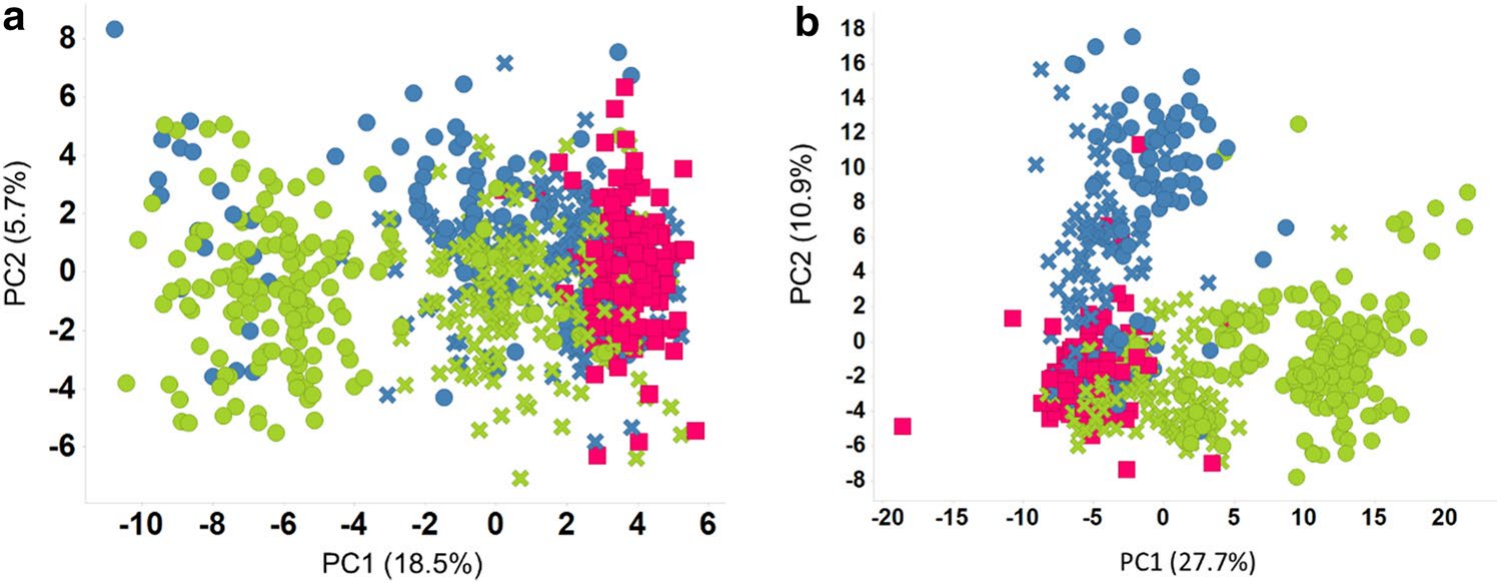
Metabolomics of specific liver MoAs. Principal component analysis (PCA) of control samples (red squares) and peroxisome proliferator-treated samples [green crosses = low dose (LD), green dots = high dose (HD); including acifluorfen, bezafibrate, dichlorprop, dichlorprop-p, fluroglycofenethyl, mecoprop, mecoprop-p and Wy-14643] as well as liver enzyme inducer-treated samples (blue crosses = LD, blue dots = HD; including aroclor, β-naphthoflavone, fipronil, pendimethalin). **a** Supernatant and **b** intracellular metabolomics data; the latter more efficiently distinguish between different MoAs and dose-dependency (color figure online)

The PCA data of supernatant and cell lysates induced by reference test substances clearly showed that the degree of distinction between the two different MoAs is superior when using intracellular metabolomics data (Fig. 5). Moreover, the intracellular metabolomics data also better reveal the concentration-dependency (LC versus HC). Considering this information, we decided to use only intracellular metabolomics data from HC treatment as the most expedient way to draw conclusions about specific toxic effects and MoAs.

First, we analyzed the metabolic profile of all substances tested to establish a general pattern of liver toxicity comprising metabolites concordantly regulated in most of the treatments. We identified 38 changed metabolites that showed “general or common liver metabolite changes” (Supplementary Fig. 1). This pattern comprised 25 lipids (including 16 unknowns), 6 energy related metabolites, 5 amino acids, 1 amino acid-related metabolite and 1 carbohydrate. Identifying these metabolites was crucial to “clean-up” the MoA profiles and to enhance their specificity.

Second, based on the metabolite profiling of at least 3 reference test substances sharing the same liver toxic MoA, we identified patterns of metabolite changes that are common for these at least three reference compounds, i.e., these metabolites are showing statistically significant similar regulation for all reference compounds used. This list of statistically significantly changed metabolites was further refined by subtracting the list of general toxicity metabolites, resulting in a more specific pattern. Now the resulting pattern was analyzed against the complete data set of all tested substances and further refined through addition or removal of metabolites in order to increase specificity and sensitivity. Specificity and sensitivity are given if the resulting pattern can identify further reference compounds with the same mode of action while at the same time excluding other compounds with a different mode of action. Applying this procedure to reference substances sharing liver enzyme induction or peroxisome proliferation MoAs enabled us to identify specific signature patterns for each MoA.

### Liver enzyme inducers (intracellular metabolome)

**Fig. 6:**
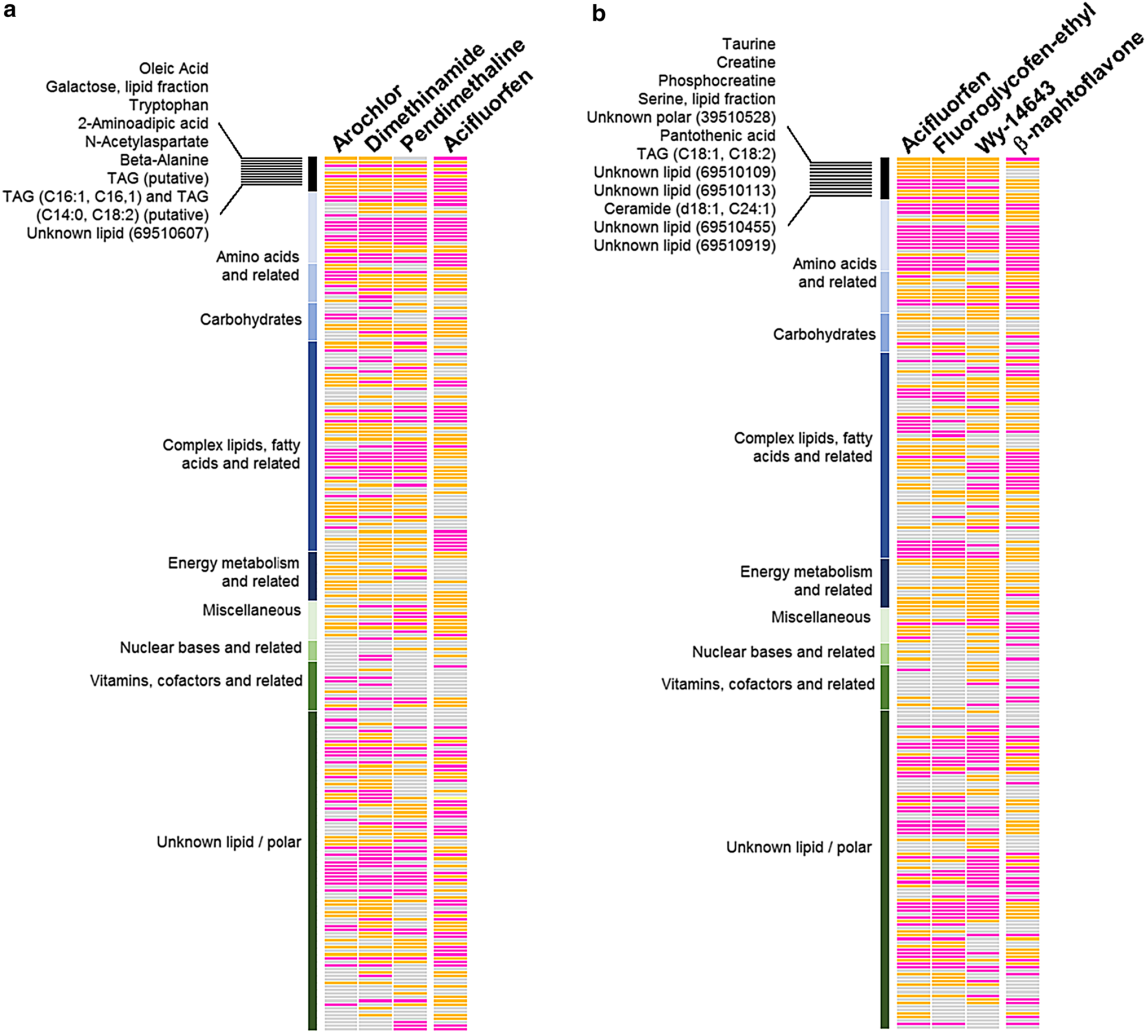
Heat map of metabolome changes induced by liver enzyme inducers and peroxisome proliferators in HepG2 cells. Yellow indicates statistically significant (*p* = 0.05) downregulation and magenta indicates statistical significant (*p* = 0.05) upregulation of the compound classes indicated; gray represent no statistical significant change. The metabolite classes are depicted in the vertical colored bars to the left of each heat map. **a** The metabolome changes induced by 3 liver enzyme inducers at HD, aroclor, dimethinamide and pendimethaline; the combination of metabolites that allow distinction of the liver enzyme inducers’ mode of action (pattern) is listed on the top left. For comparison, the metabolome changes induced by a peroxisome proliferator, acilfluorfen, are also displayed, clearly demonstrating a different pattern. **b** The metabolome changes induced by 3 peroxisome proliferators at HD, acylfluorfen, fluoroglycofen-ethyl and Wy-14643, including the list of metabolites that serve to distinguish the peroxisome proliferation mode of action (pattern). The metabolome pattern of changes induced by the liver enzyme inducer, β-naphthoflavone clearly differs from peroxisome proliferators

Using the metabolome profiles of aroclor, pendimethaline and dimethenamide, three typical liver enzyme inducers, we established a pattern comprising 9 metabolites: 3 lipid and complex lipid metabolites, 4 amino acids and related, 2 triacylglycerols (Fig. 6). With the pattern in place, metabolite profiling could identify other compounds with liver enzyme inducing properties: fipronil, dimethenamide-p, pyridaben, β-naphthoflavone and vinclozoline. Interestingly, profile comparison with a peroxisome proliferator, acifluorfen (see below), revealed a different pattern. This confirms a biological selectiveness related to the MoA of the test substances and the effects induced in the cells.

### Liver peroxisome proliferators (intracellular metabolome)

Three liver peroxisome proliferator (PP) reference substances were used to establish a PP pattern: acifluorfen, fluoroglycofen-ethyl and Wy-14643. The signature pattern contained 12 intracellular metabolites, including 4 amino acids and related, 6 lipids and complex lipids and 2 from other metabolite classes (Fig. 6). This pattern enabled us to recognize further PP substances: bezafibrate, dichlorprop, dichlorprop-p and mecroprop, indicating the reliability of the signature pattern to identify specific changes associated with peroxisome proliferators. Mecroprop-p was also correctly identified at a 0.15 *p* value; this was due to the statistical variation of one metabolite. Benzyl butyl phthalate was not identified, possibly due to its very low solubility as well as the rather low metabolizing capacity of HepG2 cells (taking into account that the phthalate monoesters are the active metabolites for phthalate toxicity). These findings represent the proof of concept that it is possible to recognize toxicological MoAs in a reliable and reproducible manner using metabolomics in vitro applied to cellular models.

To extend our metabolome analysis into a more mechanistic analysis of toxicity effects, we focused further on PPs. These include pharmaceutical and industrial chemicals that increase the number and size of peroxisomes in vivo (Corton and Lapinskas 2005), and can enhance beta oxidation, which plays an important role in lipid metabolism. During beta oxidation, peroxisomes oxidize a major proportion of very long chain fatty acids using coenzyme A, synthesized intracellularly from pantothenic acid, as an initial activator. Subsequently, bile acid-CoA thioesters are cleaved to form unconjugated bile acids and converted to bile salts by conjugation to taurine and glycine before secretion into the bile (Vessey et al. 1983; Chiang 1998). We focused on the biosynthesis of pantothenic acid and taurine, as well as changes in metabolites related to lipid metabolism (Fig. 6). Both pantothenic acid and taurine were down-regulated, possibly because the cells would need more pantothenic acid to produce acetyl CoA as an activator of beta oxidation, and require more taurine to conjugate the bile acid products of beta oxidation (Chiang 1998).

Many of the metabolites related to lipid metabolism, specifically those involved in the biosynthesis of unsaturated fatty acids, were down-regulated (i.e., eicosadienoic acid, eicosapentaenoic acid, elaidic acid), probably due to enhanced peroxisomal activity. This applied to the two classical hypolipidemic agents, Wy-14643 and bezafibrate, but was slightly different for acilfluorfen and fluoroglycofen-ethyl. The latter also induce biochemical and morphological changes in liver attributable to peroxisome proliferation (HSE 1992), but might not have such a targeted effect on lipid metabolism as the hypolipidemic agents especially designed to affect lipid metabolism.

### PCA analysis of several classes of compounds

**Fig. 7:**
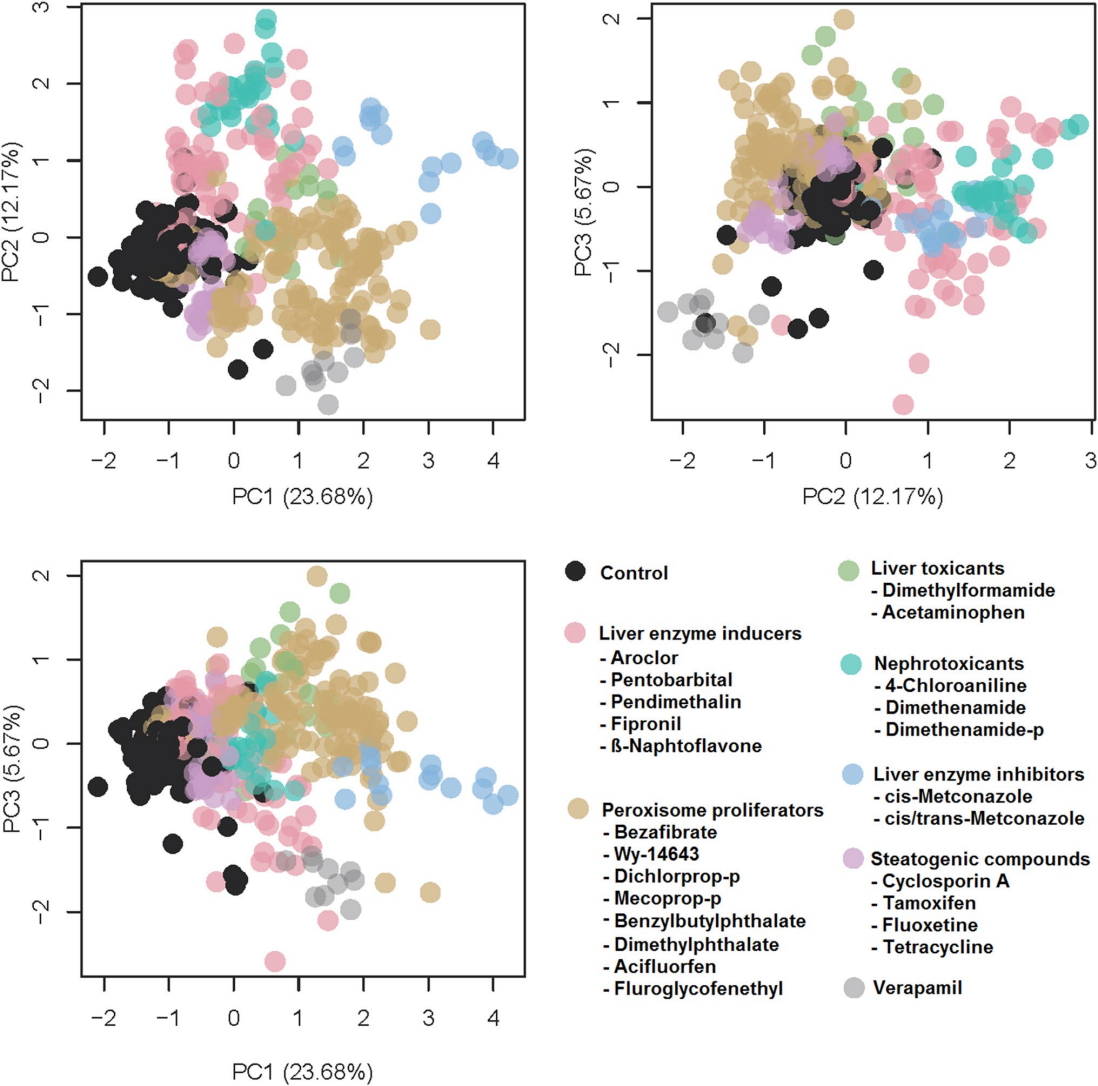
Overview of a joint PCA analysis. The plot includes liver enzyme inducers, liver enzyme inhibitors, liver toxicants, peroxisome proliferators, steatogenic compounds, nephrotoxicants and verapamil. Benzylbutylphthalate and dimethylphthalate match close to controls, since metabolic conversion to the monoesters takes not place in HepG2 cells, which is necessary for the mode of action

In the above paragraphs, we have described a procedure to identify liver MoAs by determining specific patterns of metabolite change. This is basically the same method which we have been using for the identification of systemic toxicity MoAs in in vivo studies (van Ravenzwaay et al. 2007; Kamp et al. 2012b). A different way of identifying properties of compounds is to do a PCA comparison. An overview of a normalized joint PCA analysis is shown in Fig. 7, and a detailed three-dimensional navigable graph of this PCA is shown as supplementary file, in which the single compounds can be identified. Note that benzylbutylphthalate and dimethylphthalate match close to the controls, since metabolic conversion to the monoesters takes not place in HepG2 cells, which is necessary for the mode of action.

The results of the overall PCA analysis clearly demonstrate a good separation between peroxisome proliferators, enzyme inducers, enzyme inhibition, compounds which cause liver toxicity and compounds which are not primarily liver toxic (in this case nephrotoxicants). The apparent continuum of liver enzyme induction and liver toxicity was also noted in a very similar way in the in vivo analysis of such compounds (van Ravenzwaay et al. 2012b). Thus, following the metabolome analysis of a new compound, a PCA comparison with that of reference compounds (i.e., compounds with a known MoA) may help to quickly identify the probable MoA of a new compound.

## Discussion

In the present study, we used the human hepatoma cell line HepG2 for prediction of liver toxicity and mode of action using metabolomics in vitro. HepG2 cells are easier to handle than primary human hepatocytes and are superior regarding reproducibility. However, one drawback of this cell line is its limited metabolic capacity. This applies particularly to phase I enzymes, which are expressed magnitudes lower compared to primary human hepatocytes (Wilkening et al. 2003). Therefore, compounds which require metabolic activation to exert toxic effects like acetaminophen (CYP2E1, Raucy et al. 1989) or nitrobenzodiazepines (CYP3A4, Mizuno et al. 2009) may not be detected correctly by this system, since these enzymes are only very scarcely expressed in HepG2 cells. However, this limitation could be overcome by induction of Cytochrome P450 enzymes with TCCD, ß-naphthoflavone, phenobarbital of rifampicin (Gerets et al. 2012; Garcia-Canton et al. 2013) or by using engineered HepG2 cells expressing Cytochrome P450 enzymes (Yoshitomi et al. 2001). Another limitation of the HepG2 cell line is that various nuclear receptors are expressed at a considerably lower level in these cells (Tolosa et al. 2016). Thus, compounds like phenobarbital, which acts via activation of CAR and PXR receptors, might not exhibit the complete toxicological feature compared to the in vivo situation.

Despite these limitations, we achieved standardization and reproducibility as well as the robustness of metabolomics in an in vitro human cell system. We also successfully identified, with dose–response and high specificity, different modes of action of liver toxicants (liver enzyme induction/inhibition, liver toxicity and peroxisome proliferation) by comparing metabolome profiles. For example, PPARα agonists (peroxisome proliferators in rats and mice) revealed clear changes in metabolites related to beta oxidation of fatty acids, correlating well with the mechanism of this class of test substances in the in vivo situation, and confirming the reliability of the system. Within this context, it should be mentioned that the upregulating effects of PPARα agonists on fatty acid oxidation in vivo occur in both rodents and primates, the magnitude being greater in rodents. In rats and mice, these compounds induce peroxisome proliferation, hepatocellular hypertrophy and liver tumors. In contrast, primates are much more resistant to peroxisome proliferation and hepatocellular hypertrophy, and peroxisome proliferation or increased incidence of liver tumors was not observed in humans treated chronically with fibrates (Klaunig et al. 2003). One reason why primates are refractory to liver carcinogenesis may be that apoptosis is downregulated and cell proliferation is increased in the rodent, but not in the primate liver (Hoivik et al. 2004). Based on that, the PPARα response seen in the human HepG2 cell line reflects the upregulation of fatty acid oxidation. Based on the marked species differences outlined above, the PPARα signature in HepG2 cells is unlikely to predict a human risk concerning liver cancer.

It should be noted that the purpose of this study was not a full validation of HepG2 cell-based metabolomics as a tool for liver toxicity, but more to show proof of concept that this technology can identify different liver toxicities. Therefore, no negative compounds for liver toxicity have been included. In order to achieve this goal, the compound concentrations tested here were as high as possible, but below significantly cytotoxic levels (i.e., viability > 80%) without taking into account whether these concentrations do reflect relevant in vivo plasma or tissue concentrations. This approach poses the risk that these high concentrations could saturate metabolism or detoxifying mechanisms. Therefore, the observed effects could be different from those at lower, possibly more physiological concentrations. Additionally, other mechanisms could play a role at lower concentrations.

Garcia-Cañaveras et al. examined changes in the metabolome after incubation of HepG2 cells with compounds causing no liver toxicity and compounds causing oxidative stress, steatosis and phospholiposis (García-Cañaveras et al. 2015, 2016). Using PCA analysis and PLS-DA (projection to latent structures-discriminant analysis), they found a clear separation of specifically altered metabolites for each compound class, which allowed unravelling changes in the respective underlying biochemical pathways. In this respect, our data confirm these results, but also add data of liver enzyme inducers/inhibitors, liver toxicants and peroxisome proliferators to the available database.

The advantages of combining metabolomics with an in vitro system are manifold. (1) It reduces the need for animal studies, (2) the amount of test substance needed is in the range of 100–200 mg, allowing for early screening of novel compounds, (3) measurement of intracellular metabolites provides data which can be plotted on biochemical charts, making a biochemical interpretation of the results possible, (4) connecting observed disturbances in biochemical pathways with known modes of action (MoA) will help to identify adverse outcome pathways, (5) creating patterns of metabolite changes typical for a particular MoA will lead to fast identification of the toxicological properties of new compounds, and (6) if a database is created which is large enough, comparison of the metabolome profile of a compound under investigation with all other compounds in the database will also provide important information on its toxicity as is already shown for plasma metabolomics in vivo (see references of this group). Generally, we have found that PCA comparisons are adequate in predicting the toxicological MoA of the compounds investigated.

One can envisage expanding MoA identification by selecting different reference substances exhibiting other toxic effects in the liver, as well as establishing kidney or neuronal cellular systems to enhance the battery of cellular models that can be combined with metabolomics. For example, we have previously demonstrated that rat plasma metabolomics can identify the MoA of hepatotoxic or kidney toxic compounds (van Ravenzwaay et al. 2012b; Kamp et al. 2012a; Mattes et al. 2014). A battery of human cellular models covering different organs, an “organ toxicity-toolbox” for testing chemicals over prolonged periods, would transform metabolomics in vitro into a powerful tool to accurately measure changes in these cells and rapidly predict toxicity. Moreover, combining information from different organ-like models would contribute to future risk assessment based on altering toxicity pathways, as has been proposed for the use of transcriptomics (De Abrew et al. 2015). We also have evidence for the feasibility of many other applications, such as chemical grouping (Ramirez et al. in preparation), opening new possibilities for the application of metabolomics in vitro in the regulatory area.

A further area which merits exploration is the quantitative relationship between metabolite changes in this in vitro setting and results observed in animal studies. Is there a possibility to distinguish between true adverse effects and adaptive changes at the level of metabolites? Can quantitative differences in the sensitivity of humans and rats (as the most commonly used animal model) also be seen when comparing rat and human cells when applying metabolomics to liver cells of both species? Although at this time these answers cannot be given, they become testable with the technology presented in this paper.

In conclusion, in vitro metabolomics systems can help identify organ toxicity, determine the toxicological profile of different test substances, predict the toxicity of new compounds, and better elucidate the molecular mechanisms underlying their toxicity in highly controllable systems suitable for regulatory purposes, and most importantly, without animal testing.

## Electronic supplementary material

Below is the link to the electronic supplementary material.

Supplementary material 1 (DOCX 75 kb)

Supplementary material 2 (DOCX 3261 kb)

## References

[CR1] Arrowsmith J (2012). A decade of change. Nat Rev Drug Discov.

[CR2] Attene-Ramos MS, Huang R, Michael S (2015). Profiling of the Tox21 chemical collection for mitochondrial function to identify compounds that acutely decrease mitochondrial membrane potential. Environ Health Perspect.

[CR3] Balcke GU, Kolle SN, Kamp H (2011). Linking energy metabolism to dysfunctions in mitochondrial respiration—a metabolomics in vitro approach. Toxicol Lett.

[CR4] Bordag N, Rennfahrt U, Nachtigall J (2016). Fast sampling of adherent cell cultures for optimal metabolomics results. Metabolomics.

[CR5] Bordag N, Rennfahrt U, Nachtigall J (2016). Fast filtration of bacterial or mammalian suspension cell cultures for optimal metabolomics results. PLoS One.

[CR6] Bouhifd M, Hartung T, Hogberg HT (2013). Review: toxicometabolomics. J Appl Toxicol JAT.

[CR7] Chiang JY (1998). Regulation of bile acid synthesis. Front Biosci.

[CR8] Choucha Snouber L, Bunescu A, Naudot M (2013). Metabolomics-on-a-chip of hepatotoxicity induced by anticancer drug flutamide and Its active metabolite hydroxyflutamide using HepG2/C3a microfluidic biochips. Toxicol Sci.

[CR9] Corton JC, Lapinskas PJ (2005). Peroxisome proliferator-activated receptors: mediators of phthalate ester-induced effects in the male reproductive tract?. Toxicol Sci.

[CR10] De Abrew KN, Overmann GJ, Adams RL (2015). A novel transcriptomics based in vitro method to compare and predict hepatotoxicity based on mode of action. Toxicology.

[CR11] García-Cañaveras JC, Jiménez N, Gómez-Lechón MJ (2015). LC-MS untargeted metabolomic analysis of drug-induced hepatotoxicity in HepG2 cells. Electrophoresis.

[CR12] García-Cañaveras JC, Castell JV, Donato MT, Lahoz A (2016). A metabolomics cell-based approach for anticipating and investigating drug-induced liver injury. Sci Rep.

[CR13] Garcia-Canton C, Minet E, Anadon A, Meredith C (2013). Metabolic characterization of cell systems used in in vitro toxicology testing: lung cell system BEAS-2B as a working example. Toxicol In Vitro.

[CR14] Gerets HH, Tilmant K, Gerin B (2012). Characterization of primary human hepatocytes, HepG2 cells, and HepaRG cells at the mRNA level and CYP activity in response to inducers and their predictivity for the detection of human hepatotoxins. Cell Biol Toxicol.

[CR15] Hartung T (2009). Toxicology for the twenty-first century. Nature.

[CR16] Hoivik DJ, Qualls CW, Mirabile RC (2004). Fibrates induce hepatic peroxisome and mitochondrial proliferation without overt evidence for cellular proliferation and oxidative stress in cynomolgus monkeys. Carcinogenesis.

[CR17] HSE (1992). Evaluation on: fluoroglycofen-ethyl. May 1992. Issue No. 50. Department for Environment, Food and Rural Affairs.

[CR18] Judson R, Houck K, Martin M (2014). In vitro and modelling approaches to risk assessment from the US Environmental Protection Agency ToxCast programme. Basic Clin Pharmacol Toxicol.

[CR19] Jung K, Reszka R, Kamlage B (2013). Tissue metabolite profiling identifies differentiating and prognostic biomarkers for prostate carcinoma. Int J Cancer.

[CR20] Kamp H, Fabian E, Groeters S (2012). Application of in vivo metabolomics to preclinical/toxicological studies: case study on phenytoin-induced systemic toxicity. Bioanalysis.

[CR21] Kamp H, Strauss V, Wiemer J (2012). Reproducibility and robustness of metabolome analysis in rat plasma of 28-day repeated dose toxicity studies. Toxicol Lett.

[CR22] Klaunig JE, Babich MA, Baetcke KP (2003). PPAR alpha agonist-induced rodent tumors: modes of action and human relevance. Crit Rev Toxicol.

[CR23] Liu J, Mansouri K, Judson RS (2015). Predicting hepatotoxicity using ToxCast in vitro bioactivity and chemical structure. Chem Res Toxicol.

[CR24] Mattes W, Davis K, Fabian E (2014). Detection of hepatotoxicity potential with metabolite profiling (metabolomics) of rat plasma. Toxicol Lett.

[CR25] Mizuno K, Katoh M, Okumura H (2009). Metabolic activation of benzodiazepines by CYP3A4. Drug Metab Dispos.

[CR26] Olson H, Betton G, Robinson D (2000). Concordance of the toxicity of pharmaceuticals in humans and in animals. Regul Toxicol Pharmacol RTP.

[CR27] Ramirez T, Ramirez T, Ahlbory-Dieker D (2012). Identification of liver toxicity by combining in vitro cell systems and metabolomics. Toxicol Lett.

[CR28] Ramirez T, Bordag N, Mellert W (2013). Application of metabolomics in vitro for identification of toxicological modes of action. Toxicol Lett.

[CR29] Ramirez T, Daneshian M, Kamp H (2013). Metabolomics in toxicology and preclinical research. Altex.

[CR30] Raucy JL, Lasker JM, Lieber CS, Black M (1989). Acetaminophen activation by human liver cytochromes P450IIE1 and P450IA2. Arch Biochem Biophys.

[CR31] Reily MD, Tymiak AA (2015). Metabolomics in the pharmaceutical industry. Drug Discov Today Technol.

[CR32] Tolosa L, Gomez-Lechon MJ, Lopez S (2016). Human upcyte hepatocytes: characterization of the hepatic phenotype and evaluation for acute and long-term hepatotoxicity routine testing. Toxicol Sci.

[CR33] Van den Hof WFPM, Van Summeren A, Lommen A (2014). Integrative cross-omics analysis in primary mouse hepatocytes unravels mechanisms of cyclosporin A-induced hepatotoxicity. Toxicology.

[CR34] van Ravenzwaay B, Cunha GC-P, Leibold E (2007). The use of metabolomics for the discovery of new biomarkers of effect. Toxicol Lett.

[CR35] van Ravenzwaay B, Brunborg G, Kleinjans J (2012). Use of’omics to elucidate mechanism of action and integration of’omics in a systems biology concept. Mutat Res.

[CR36] van Ravenzwaay B, Herold M, Kamp H (2012). Metabolomics: a tool for early detection of toxicological effects and an opportunity for biology based grouping of chemicals-from QSAR to QBAR. Mutat Res.

[CR37] Vermeersch KA, Wang L, Mezencev R (2015). OVCAR-3 spheroid-derived cells display distinct metabolic profiles. PLoS One.

[CR38] Vessey DA, Whitney J, Gollan JL (1983). The role of conjugation reactions in enhancing biliary secretion of bile acids. Biochem J.

[CR39] Walk TB, Dostler M (2003) Mass spectrometry method for analysing mixtures of substances. WO2003073464 A1, p. PCT/EP2003/001274, Patent WO2003073464

[CR40] Wilkening S, Stahl F, Bader A (2003). Comparison of primary human hepatocytes and hepatoma cell line HepG2 with regard to their biotransformation properties. Drug Metab Dispos.

[CR41] Yoshitomi S, Ikemoto K, Takahashi (2001). Establishment of the transformants expressing human cytochrome P450 subtypes in HepG2, and their applications on drug metabolism and toxicology. Toxicol In Vitro.

