# Supplementary material for: Prediction of liver toxicity and mode of action using metabolomics in vitro in HepG2 cells
Source: Arch Toxicol. 2017 Sep 30;92(2):893–906. doi: 10.1007/s00204-017-2079-6 (PMC5818600; doi:10.1007/s00204-017-2079-6)

**Supplementary data**

**Supplementary Table 1. Overview of the test substances used for treatment of HepG2 cells for 48 h.** Those highlighted in gray represent compounds discussed in the text.

| **Substance** | **CAS-Nr.** | **Supplier** | **MW g/mol** | **Chemical class** | **Category** | **MoA (target in)** | **Source** |
| --- | --- | --- | --- | --- | --- | --- | --- |
| **4-Chloroaniline** | 106-47-8 | Sigma-Aldrich | 127.57 | Amine | Industrial chemical | Methemoglobin formation | PubmedChem Compound, 2015 |
| **β-Naphthoflavone** | 6051-87-2 | Sigma-Aldrich | 272.29 | Benzoflavone | Industrial chemical | Liver enzyme inducer | Johnson et al., 1994 |
| **Acetaminophen** | 103-90-2 | Sigma-Aldrich | 151.16 | Drug | Pharma | Cyclooxygenase inhibitor | Graham and Scott, 2005 |
| **Acifluorfen** | 50594-66-6 | Sigma-Aldrich | 361.66 | Diphenylether | Herbicide | Inhibition of protoporphyrinogen oxidase (PPO) | [http://weedscience.org](http://weedscience.org/) |
| **Aroclor 1254** | 11097-69-1 | Chem Service | 326.43 | Polychlorinated biphenyl | Industrial chemical | Liver enzyme inducer | PubmedChem Compound, 2015 |
| **Benzyl butyl phthalate** | 85-68-7 | Sigma-Aldrich | 312.35 | Phthalic acids | Industrial chemical | Peroxisome proliferation | PubmedChem Compound, 2015 |
| **Bezafibrate** | 41859-67-0 | Sigma-Aldrich | 361.81 | Fibric acids | Hypolipidemic agents | Peroxisome proliferation | Drug Bank (DB01393) |
| **Carbaryl** | 63-25-2 | Sigma-Aldrich | 201.22 | Carbamate | Insecticide | Acetylcholinesterase (AChE) inhibitors | [www.irac-online.org](http://www.irac-online.org/) |
| **Cyclosporin A** | 59865-13-3 | Sigma-Aldrich | 1202.61 | Peptides, cyclic | Immunosuppresive agents | block the transcription of cytokine genes in activated T cells | Drug Bank (DB00091); Matsuda and Koyasu, 2000 |
| **Cycloxidim** | 101205-02-1 | Sigma-Aldrich | 325.46 | Pyrans | Herbicide | Fatty acid biosynthesis in grass | Pubmed Compound 2015; http://weedscience.org |
| **Dichlorprop** | 120-36-5 | BASF | 235.06 | Phenoxyacetate | Herbicide | Action like indole acetic acid (synthetic auxins) | Pubmed Compound 2015; http://weedscience.org |
| **Dichlorprop-p** | 15165-67-0 | BASF | 235.06 | Phenoxyacetate | Herbicide | Action like indole acetic acid (synthetic auxins) | Pubmed Compound 2015; http://weedscience.org |
| **Digitoxin** | 71-63-6 | Sigma-Aldrich | 764.93 | Digitalis glycosides | Anti-arrhythmia agent | Heart / Na-K ATPase inhibitor | HSDB, 2015 |
| **Dimethenamide** | 87674-68-8 | BASF | 275.79 | Chloroacetamide | Herbicide | Long chain fatty acid inhibitor | Pubmed Compound, 2015; Goetz and Boeger, 2004 |
| **Dimethenamide-p** | 163515-14-8 | BASF | 275.79 | Chloroacetamide | Herbicide | Long chain fatty acid inhibitor | Pubmed Compound, 2015 |
| **Dimethoate** | 60-51-5 | BASF | 229.25 | Organophosphate | Insecticide | Acetylcholinesterase (AChE) inhibitors | [www.irac-online.org](http://www.irac-online.org/) |
| **Dimethylformamide** | 68-12-2 | Sigma-Aldrich | 73.09 | Formamide | Industrial chemical | Not applicable (liver toxicant) | PubmedChem Compound, 2015 |
| **Fipronil** | 120068-37-3 | BASF | 437.14 | Phenylpyrazole | Insecticide | GABA -gated chloride channel blockers | www.irac-online.org |
| **Fluoroglycofen-ethyl** | 77501-90-7 | Sigma-Aldrich | 447.74 | Diphenylether | Herbicide | Inhibition of protoporphyrinogen oxidase (PPO) | Pubchem Compound, 2015; http://weedscience.org |
| **Fluoxetine hydrochloride** | 56296-78-7 | BASF | 345.78 | Propyolamine | Antidepressant | selective serotonin reuptake inhibitor | Pubchem Compound, 2015; Perez-Caballero et al., 2014 |
| **Imazamox** | 114311-32-9 | BASF | 305.32 | Imidazole | Herbicide | Inhibition of acetolactate synthase ALS (acetohydroxyacid synthase AHAS) | http://weedscience.org |
| **MCPA** | 94-74-6 | Rhone-Poulenc | 200.61 | Phenoxyacetate | Herbicide | Action like indole acetic acid (synthetic auxins) | [http://weedscience.org](http://weedscience.org/) |
| **Mecroprop** | 93-65-2 | BASF | 214.64 | Phenoxyacetate | Herbicide | Action like indole acetic acid (synthetic auxins) | Pubchem Compound, 2015; http://weedscience.org |
| **Mecoprop-p** | 16484-77-8 | BASF | 214.64 | Phenoxyacetate | Herbicide | Action like indole acetic acid (synthetic auxins) | Pubchem Compound, 2015; http://weedscience.org |

| **Metconazole/cis** | 115850-27-6 | BASF | 319.83 | Triazole derivative | Fungicide | Enzyme Inhibitor | US EPA Pesticide Fact Sheet, 2007; https://www3.epa.gov/pesticides/chem_search/reg_actions/registration/fs_PC-125619_01-Sep-07.pdf |
| --- | --- | --- | --- | --- | --- | --- | --- |
| **Metconazole /cis-trans** | 125116-23-6 | BASF | 319.83 | Triazole derivative | Fungicide | Enzyme Inhibitor | US EPA Pesticide Fact Sheet, 2007; https://www3.epa.gov/pesticides/chem_search/reg_actions/registration/fs_PC-125619_01-Sep-07.pdf |
| **Nicosulfuron** | 111991-09-4 | Sigma-Aldrich | 410.40 | Sulfonylurea | Herbicide | Inhibition of acetolactate synthase ALS (acetohydroxyacid synthase AHAS) | Pubmed Compound, 2015; http://weedscience.org |
| **Pendimethalin** | 40487-42-1 | BASF | 281.3 | Dinitroaniline | Herbicide | Microtubule assembly inhibition | <http://weedscience.org/documents/showdocuments.aspx?DocumentID=1192> |
| **Pentobarbital sodium salt** | 57-33-0 | Sigma-Aldrich | 248.25 | Barbiturate | Sedative | Brain/GABA modulator | HSDB, 2015 |
| **Pyridaben** | 96489-71-3 | BASF | 364.93 | Pyridazine | Pesticide | Mitochondrial complex I electron transport inhibitors | www.irac-online.org; Gomez et al., 2007 |
| **Tamoxifen** | 10540-29-1 | Sigma-Aldrich | 371.51 | Stilbenes | Antineoplastic | Estrogen receptor modulator | HSDB, 2015 |
| **Tetracycline** | 60-54-8 | Sigma-Aldrich | 444.43 | Tetracyclines | Pharma | Protein synthesis inhibitor | Nguyen et al., 2014 |
| **Verapamil hydrochloride** | 152-11-4 | Sigma-Aldrich | 491.06 | Phenethylamine | Anti-arrhythmia agent | Heart/Ca2+ channel blocker / CYP3A4 inhibitor | HSDB, 2015; Richards, 2005 |
| **Vinclozolin** | 50471-44-8 | BASF | 286.11 | Oxazoles | Fungicide | NADH cytochrome c reductase in lipid peroxidation | van Ravenzwaay et al., 2013, Kavlock and Cummings, 2005 |
| **Wy-14643** | 50892-23-4 | Sigma-Aldrich | 323.79 | Pyrimidines | Hypolipidemic agents | Peroxisome proliferation | Gonzalez and Shah, 2008; Suga, 2004 |

**Supplementary Table 2. Overview of the experiments per test substance.** The data indicate the test substance concentrations used as well as the effects on protein concentration (BCA in percentage) and viability (WST-1) in HepG2 cells treated for 48 h. Rows highlighted in gray represent compounds discussed in the text; the BCA % values highlighted in speckled gray indicate values under 70%. n.a.= not applicable.

µM= micro molar; n.a. = not assessed; *= LD/HD; **= only measured in experiments 1 and 2.

| **Substance** | **CAS-Nr.** | **Year** | **Experiment Reference** | **Replicates** | **Low Dose (LD)** | | | **High Dose (HD)** | | | | | |
| --- | --- | --- | --- | --- | --- | --- | --- | --- | --- | --- | --- | --- | --- |
|  |  |  |  |  | **Concentration µM** | **BCA %** | **WST-1 %** | | | **Concentration µM** | **BCA %** | **WST-1 %** |  |
| **4-Chloroaniline** | 106-47-8 | 2013 | 2 | 10 | 20 | 73 | 93 | | | 60 | 67 | 90 |  |
| **β-Naphthoflavone** | 6051-87-2 | 2015 | 5 | 8 | 10 | 96 | n.a. | | | 30 | 92 | n.a. |  |
| **Acetaminophen** | 103-90-2 | 2012 | 1 | 10 | 1800 | 92 | 98 | | | 5400 | 79 | 92 |  |
| **Acifluorfen** | 50594-66-6 | 2015 | 5 | 8 | 66.7 | 104 | n.a. | | | 200 | 92 | n.a. |  |
| **Aroclor 1254** | 11097-69-1 | 2012, 2013 | 1, 2 | 50 | 50 | 91 | 102 | | | 75 | 69 | 105 |  |
| **Benzyl butyl phthalate** | 85-68-7 | 2015 | 5 | 8 | 25 | 99 | n.a. | | | 75 | 94 | n.a. |  |
| **Bezafibrate** | 41859-67-0 | 2012-2015 | 1 to 5 | 148/276* | 333 | 86 | 98** | | | 1000 | 68 | 97** |  |
| **Carbaryl** | 63-25-2 | 2013 | 2 | 10 | 33.3 | 101 | 103 | | | 100 | 84 | 105 |  |
| **Cyclosporin A** | 59865-13-3 | 2015 | 5 | 8 | 0.33 | 94 | n.a. | | | 1 | 91 | n.a. |  |
| **Cycloxidim** | 101205-02-1 | 2013 | 2 | 10 | 200 | 86 | 105 | | | 600 | 75 | 106 |  |
| **Dichlorprop** | 120-36-5 | 2013 | 3 | 8 | 333 | 91 | n.a. | | | 1000 | 62 | n.a. |  |
| **Dichlorprop-p** | 15165-67-0 | 2013 | 3 | 8 | 333 | 87 | n.a. | | | 1000 | 69 | n.a. |  |
| **Digitoxin** | 71-63-6 | 2013 | 2 | 10 | 0.0067 | 96 | 97 | | | 0.02 | 74 | 87 |  |
| **Dimethenamide** | 87674-68-8 | 2013 | 3 | 8 | 50 | 95 | n.a. | | | 150 | 91 | n.a. |  |
| **Dimethenamide-p** | 163515-14-8 | 2013 | 3 | 8 | 50 | 97 | n.a. | | | 150 | 90 | n.a. |  |
| **Dimethoate** | 60-51-5 | 2013 | 2 | 10 | 500 | 94 | 100 | | | 1500 | 81 | 96 |  |
| **Dimethylformamide** | 68-12-2 | 2012 | 1 | 10 | 69000 | 83 | 107 | | | 138000 | 72 | 67 |  |
| **Fipronil** | 120068-37-3 | 2015 | 5 | 8 | 5 | 96 | n.a. | | | 15 | 91 | n.a. |  |
| **Fluoroglycofen-ethyl** | 77501-90-7 | 2015 | 5 | 8 | 40 | 105 | n.a. | | | 120 | 92 | n.a. |  |
| **Fluoxetine hydrochloride** | 56296-78-7 | 2015 | 5 | 8 | 2.5 | 91 | n.a. | | | 7.5 | 81 | n.a. |  |
| **Imazamox** | 114311-32-9 | 2014 | 4 | 8 | 10 | 101 | n.a. | | | 50 | 101 | n.a. |  |
| **MCPA** | 94-74-6 | 2014 | 4 | 8 | 250 | 91 | n.a. | | | 750 | 78 | n.a. |  |
| **Mecroprop** | 93-65-2 | 2013 | 3 | 8 | 333 | 90 | n.a. | | | 1000 | 69 | n.a. |  |
| **Mecoprop-p** | 16484-77-8 | 2013 | 3 | 8 | 333 | 92 | n.a. | | | 1000 | 79 | n.a. |  |
| **Metconazole/cis** | 115850-27-6 | 2013 | 3 | 8 | 10 | 90 | n.a. | | 30 | | 58 | n.a. | |
| **Metconazole /cis-trans** | 125116-23-6 | 2013 | 3 | 8 | 10 | 80 | n.a. | | 30 | | 50 | n.a. | |
| **Nicosulfuron** | 111991-09-4 | 2014 | 4 | 8 | 250 | 96 | n.a. | | | 750 | 97 | n.a. |  |
| **Pendimethalin** | 40487-42-1 | 2015 | 5 | 8 | 16.7 | 100 | n.a. | | | 50 | 88 | n.a. |  |
| **Pentobarbital sodium salt** | 57-33-0 | 2012 | 1 | 10 | 375 | 91 | 83 | | | 750 | 82 | 86 |  |
| **Pyridaben** | 96489-71-3 | 2014 | 4 | 8 | 0.001 | 100 | n.a. | | | 0.005 | 80 | n.a. |  |
| **Tamoxifen** | 10540-29-1 | 2015 | 5 | 8 | 2 | 100 | n.a. | | | 6 | 89 | n.a. |  |
| **Tetracycline** | 60-54-8 | 2015 | 5 | 8 | 20 | 99 | n.a. | | | 60 | 86 | n.a. |  |
| **Verapamil hydrochloride** | 152-11-4 | 2013 | 2 | 10 | 13.3 | 82 | 99 | | | 40 | 62 | 90 |  |
| **Vinclozolin** | 50471-44-8 | 2014 | 4 | 8 | 25 | 95 | n.a. | | | 100 | 75 | n.a. |  |
| **Wy-14643** | 50892-23-4 | 2012 | 1 | 10 | 156.5 | 85 | 103 | | | 313 | 64 | 91 |  |

Supplementary Figure 1. **Heat map of metabolome changes induced by exemplary test substances tested in HepG2 cells.** Yellow indicates statistically significant (p=0.05) downregulation and magenta indicates statistical significant (p=0.05) upregulation of the indicated metabolites; grey depict no statistical significant change. Displayed are the metabolome changes induced by HD of the indicated compounds. Listed individually on the left are the metabolites that form part of a general liver toxicity pattern; subtraction of these metabolites enhances the specificity of MoA profiles.


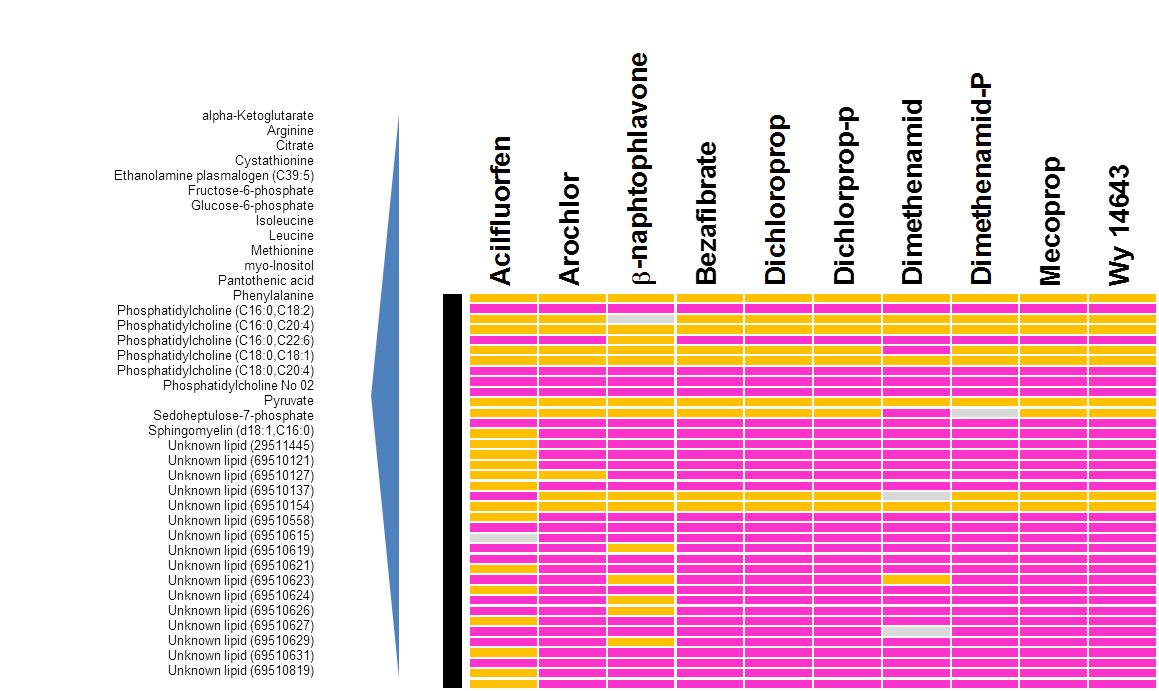

Supplement: Supplementary file 1 — Supplementary material 1 (DOCX 75 kb) [file 204_2017_2079_MOESM1_ESM.docx]
