# Supplementary material for: Prediction of liver toxicity and mode of action using metabolomics in vitro in HepG2 cells
Source: Arch Toxicol. 2017 Sep 30;92(2):893–906. doi: 10.1007/s00204-017-2079-6 (PMC5818600; doi:10.1007/s00204-017-2079-6)
Supplement: Supplementary file 2 — Supplementary material 2 (DOCX 3261 kb) [file 204_2017_2079_MOESM2_ESM.html]

3D-PCA (normalised data)


3D-PCA (normalised data)

---
